# Sulfur and Calcium Simultaneously Regulate Photosynthetic Performance and Nitrogen Metabolism Status in As-Challenged *Brassica juncea* L. Seedlings

**DOI:** 10.3389/fpls.2018.00772

**Published:** 2018-06-19

**Authors:** Rachana Singh, Parul Parihar, Sheo M. Prasad

**Affiliations:** Ranjan Plant Physiology and Biochemistry Laboratory, Department of Botany, University of Allahabad, Allahabad, India

**Keywords:** chlorophyll *a* fluorescence, glutamate dehydrogenase, glutamate synthase, glutamine synthetase, nitrate reductase, nitrite reductase, OJIP transient

## Abstract

In the present study, the role of sulfur (K_2_SO_4_: S; 60 mg S kg^-1^ sand) and/or calcium (CaCl_2_: Ca; 250 mg Ca kg^-1^ sand) applied alone as well as in combination on growth, photosynthetic performance, indices of chlorophyll *a* fluorescence, nitrogen metabolism, and protein and carbohydrate contents of Indian mustard (*Brassica juncea* L.) seedlings in the absence and presence of arsenic (Na_2_HAsO_4_.7H_2_O: As_1_; 15 mg As kg^-1^ sand and As_2_; 30 mg As kg^-1^ sand) stress was analyzed. Arsenic with its rising concentration negatively affected the fresh weight, root/shoot ratio, leaf area, photosynthetic pigments content, photosynthetic oxygen yield, and chlorophyll *a* fluorescence parameters: the O–J, J–I and I–P rise, Q_A_^-^ kinetic parameters, i.e., ΦP_0_, Ψ_0_, ΦE_0_, and PI_ABS_, along with F_v_/F_0_ and Area while increased the energy flux parameters, i.e., ABS/RC, TR_0_/RC, ET_0_/RC, and DI_0_/RC along with F_0_/F_v_ and *S*_m_ due to higher As/S and As/Ca ratio in test seedlings; however, exogenous application of S and Ca and their combined effect notably counteracted on As induced toxicity on growth and other important growth regulating processes. Moreover, inorganic nitrogen contents, i.e., nitrate (NO_3_^-^) and nitrite (NO_2_^-^) and the activities of nitrate assimilating enzymes, viz., nitrate reductase (NR) and nitrite reductase (NiR) and ammonia assimilating enzymes, viz., glutamine synthetase (GS) and glutamate synthase (GOGAT) along with protein and carbohydrate contents were severely affected with As toxicity; while under similar condition, ammonium (NH_4_^+^) content and glutamate dehydrogenase (GDH) activity in both root and leaves showed reverse trend. Furthermore, S and Ca supplementation alone and also in combination to As stressed seedlings ameliorated these parameters except NH_4_^+^ content and GDH activity, which showed an obvious reduction under similar conditions. These findings point out that exogenous application of S and/or Ca particularly S+Ca more favorably regulated the photosynthesis, contents of protein, carbohydrate and inorganic nitrogen, and the activities of nitrate and ammonia assimilating enzymes, which might be linked with the mitigation of As stress. Our results suggest that exogenous application of S+Ca more efficiently defends *Brassica* seedlings by declining As accumulation in root and shoot tissues and by maintaining the photosynthesis and nitrogen metabolism as well.

## Introduction

By 2050, the target of raising 70% agricultural productivity to feed about 2.3 billion people is facing complex challenges due to biotic and abiotic stresses (Food and Agricultural Organization [FAO], 2009), which negatively affect crop productivity. In recent decades, arsenic (As) contamination is receiving attention especially in South-East Asia including India and Bangladesh (Singh et al., 2015), where the situation has become fatal as its lower doses causes skin cancer and at higher doses it may lead to the death (Kumar et al., 2015). Through its different sources (pyrite ores, pesticides, phosphate fertilizers, mining and smelting, semi-conductors, coal combustion, etc.), arsenic is frequently released into water bodies, which is used for the irrigation of crops and fodder and thereby it enters into food chain (Gupta and Ahmad, 2014). In Indian continent, the soil concentration of As ranges from 3.34 to 105 mg kg^-1^ on contaminated sites (Patel et al., 2005). It principally exists in two inorganic forms, i.e., Arsenate (As^V^) and arsenite (As^III^); As^V^ enters in root cells through phosphate transporters and replaces phosphate in ATP metabolism, resulting into the interruption of energy flow in cells, while As^III^ enters via aquaporin channels and hampers cellular functioning by interfering with sulfhydryl (–SH) group of enzymes, their cofactors, and proteins and causes cell death (Singh et al., 2016). Once after entrance in plant, As negatively affects plant growth, water and nutrients uptake, leaf gas exchange, chlorophyll biosynthesis, photosynthetic efficiency, chlorophyll *a* fluorescence, and ribulose 1,5-bisphosphate carboxylase/oxygenase (RuBisCo) activity, which in turn reduces the growth of plants (Stoeva and Bineva, 2003; Liu et al., 2007; Singh et al., 2013; Gupta and Ahmad, 2014). In addition, enhanced level of As also negatively affects nitrogen metabolism thereby affecting inorganic nitrogen contents and activities of nitrate and ammonia assimilating enzymes (Singh et al., 2009). The As^V^ most commonly induces reactive oxygen species (ROS) generation via conversion of As^V^ into As^III^ that leads to electrons leakage from electron transport chain to O_2_, thereby causing a great loss to the integrity of cell membranes, DNA, lipids, and proteins, which are the backbone of plant cell functioning (Singh et al., 2013). To relieve As toxicity, plants have evolved antioxidant defense system (Liu et al., 2007; Singh et al., 2013; Mishra et al., 2016).

So, in order to cope up with the problems derived from As toxicity, different mineral nutrients have been frequently introduced by workers. In this chain, sulfur (S) and calcium (Ca) being an important signaling agent have been studied so far for their renowned role in plants. Sulfur is the major component of iron–sulfur (Fe–S) clusters, sulfo-lipids, vitamins (biotin and thiamine), glucosinolates, proteins, cysteine (Cys), glutathione (GSH), methionine, and thioredoxin system that regulate the physiological processes of plants (Khan and Khan, 2014). Besides, it also shares major role in build-up of photosynthetic apparatus and electron transport system and in coenzymes and prosthetic groups like ferredoxin that are vital for nitrogen assimilation (Marschner, 1995) and its deficiency directly interferes with the photosynthetic efficiency thereby influencing the activity of RuBisCo (Lunde et al., 2008). However, due to drastic reduction in the rate of S emission into atmosphere and excessive use of NPK fertilizers, only a trace amount of S is being added in the soil thereby leading to deficiency of S in soils. Therefore, sufficient amount of S is not only needed to maintain the status of plant metabolism but also in developing tolerance in plants against heavy metals in order to detoxify them (Liang et al., 2016).

Besides S, calcium (Ca) enriched growth condition could be another approach in reducing As toxicity of plants. Special attention has been paid to Ca, as it regulates cytoplasmic streaming, cell division and elongation, photomorphogenesis, and its protective role against environmental stresses (Kader and Lindberg, 2010). Additionally, Ca is a renowned secondary messenger and plays important role in cell metabolism and in the nutrients’ absorption across the cell membranes (Ahmad et al., 2015; Singh et al., 2018). The Ca has also proven to improve the photosynthetic electron transport, activities of key enzymes of Calvin cycle, and antioxidant capacity of stressed plants (You et al., 2002; Ahmad et al., 2015, 2016; Hochmal et al., 2015; Singh et al., 2018).

Indian mustard (*Brassica juncea* L.) of Brassicaceae family is ranked third among oil seed crop of world after soybean and palm. It is receiving attention due to its phytochemical content and medicinal properties. The residues of the plant are often used as cattle feed, biofuels, and fertilizer for the soil. The plant of *B. juncea* is an ideal system for evaluating metal toxicity as it is a well-known metal hyper-accumulator. Most of the studies regarding involvement of mineral nutrients in stress tolerance in plants were either dealing with S or Ca; however, no such study regarding their simultaneous effect (a prevailing condition of fields) on photosynthetic performance and status of nitrogen metabolism was reported. Therefore, present study was undertaken to evaluate the regulatory action of S and Ca individually and/or in combination in As-challenged *B. juncea* L. seedlings. Moreover, to get deeper insight of the present study, growth, As accumulation, photosynthetic pigments content, photosynthetic performance, and status of inorganic nitrogen contents and nitrogen assimilating enzymes were examined.

## Materials and Methods

### Plant Material and Growth Conditions

To carry out the present study, seeds of *B. juncea* L. were procured from local market branded as Green India Hybrid seeds Pvt. Ltd. Faizabad, Uttar Pradesh, India, and sterilized with sodium hypochlorite solution (2%, v/v) for 10 min. After that, seeds were repeatedly washed with distilled water following their wrapping in muslin cloth and kept in darkness for germination at 25 ± 2°C. After 48 h, germinated seeds were sown in plastic cups having 150 g acid washed sand and kept in darkness. After 48 h, seedlings were shifted and allowed to grow in plant growth chamber (CDR model GRW-300 DGe, Athens) having a daily cycle of 16:8 h light:dark photoperiod with photosynthetically active radiation (PAR): 150 μmol photons m^-2^ s^-1^ and relative humidity: 65–70% at 22 ± 2°C. On alternate days, seedlings were irrigated with 50% Hoagland solution (Hoagland and Arnon, 1950). Thereafter, 30-days-old seedlings were treated with As (Na_2_HAsO_4_.7H_2_O), S (K_2_SO_4_), and Ca (CaCl_2_) by dissolving the respected compounds in Hoagland nutrient solution. The experimental design consisted of: control (untreated seedlings), As_1_ (15 mg As kg^-1^ sand), As_2_ (30 mg As kg^-1^ sand), S (60 mg S kg^-1^ sand), As_1_+S, As_2_+S, Ca (250 mg Ca kg^-1^ sand), As_1_+Ca, As_2_+Ca, S+Ca, As_1_+S+Ca, and As_2_+S+Ca. It should be noted that the doses of As, S, and Ca used to carry out the experimental work in the present study were selected on the basis of screening experiments. Different parameters were analyzed after 7 days of treatment, on the root and shoot (stem+leaves)/leaves of test seedlings.

### Growth Attributes

Growth was measured in terms of fresh weight (FW), root/shoot ratio, and leaf area of the seedlings. For the measurement of FW, untreated and treated seedlings were carefully uprooted, washed with distilled water (to remove the sand particles), dried with tissue paper, and then the weight was taken using single pan digital electronic balance (Model CA 223, Contech, India). The root/shoot ratio was calculated as: root FW/ shoot FW. Leaf area was measured using leaf area meter (Systronics, India).

### Metal Accumulation

As, S, and Ca contents in root and shoot of test seedlings was assayed following the method of Allen et al. (1986).

### Photosynthetic Pigments Content

The photosynthetic pigments content were assayed by the method of Lichtenthaler (1987) by recording the absorbance at 663, 646, and 470 nm in spectrophotometer (Shimadzu double beam UV–Visible spectrophotometer-1700, Japan).

### Estimation of Photosynthesis and Respiration

Photosynthetic oxygen yield and respiratory oxygen uptake rates were performed in terms of oxygen evolution/consumption in leaf discs from untreated and treated samples in the presence and absence of light, respectively, by the method of Kurra-Hotta et al. (1987) using Clark type oxygen electrode (Digital Oxygen System, Model-10, Rank Brothers, United Kingdom).

### Measurement of Chlorophyll *a* Fluorescence Transient

Polyphasic Chl *a* fluorescence transients in 30 min dark adapted leaves (for the complete oxidation of reaction centers) was carried out using leaf fluorometer (FluorPen FP 100, Photon System Instrument, Czechia) following the method of Strasser et al. (2000). The shape of J–I–P rise is associated with the complexity of reduction kinetic of PS II acceptor side. The O–J agrees with the photochemical reduction of primary electron acceptor of PS II (Q_A_), J–I corresponds to complete closure of PS II reaction center or Q_B_ quenching mechanism, and I–P is related to reduction of PQ pool and pool size of final PS I electron acceptor. The quantum yield of primary photochemistry (ΦP_0_ or Phi_P_0_), yield of electron transport per trapped exciton (Ψ_0_ or Psi__0_), quantum yield of electron transport (ΦE_0_ or Phi_E_0_), performance index (PI_ABS_) of PS II, the energy fluxes for absorption of photon per active reaction center (ABS/RC), trapped energy flux per active reaction center (TR_0_/RC), electron transport flux per active reaction center (ET_0_/RC), energy dissipation flux per active reaction center (DI_0_/RC), size and number of active reaction centers (F_v_/F_0_), efficiency of water splitting complex (F_0_/F_v_), energy necessary for the closure of all reaction centers (*S*_m_), and the area above the fluorescence induction curve between F_0_ and F_m_ (Area) were analyzed.

### Estimation of Inorganic Nitrogen Contents

#### Nitrate (NO_3_^-^), Nitrite (NO_2_^-^), and Ammonium (NH_4_^+^) Contents

The assay of nitrate content is based on nitration of salicylic acid in acidic conditions, following the method proposed by Cataldo et al. (1975). Absorbance of the solution was recorded at 410 nm and nitrate content in the solution was calculated with the help of calibration curve prepared by KNO_3_ and expressed in terms of μmol NO_3_^-^ g^-1^ FW.

Nitrite content was assayed following the method of Snell and Snell (1949). The absorbance of the reaction mixture was recorded at 540 nm and the nitrite content in the solution was calculated with the help of standard curve prepared by KNO_2_ and expressed in terms of μmol NO_2_^-^ g^-1^ FW.

Ammonium content was assayed following the method of Molins-Legua et al. (2006) using the Nessler reagent. The absorbance of the reaction mixture was recorded at 425 nm and ammonium content was calculated with the help of calibration curve prepared by NH_4_Cl and expressed as μmol NH_4_^+^ g^-1^ FW.

### Estimation of Nitrogen Assimilating Enzymes

#### Nitrate Reductase (NR) and Nitrite Reductase (NiR) Activities

The assay of nitrate reductase (NR; EC 1.6.6.1) activity was based on the total nitrite formed by the method of Robin (1979). The absorbance of reaction mixture was recorded at 540 nm, and the content of NO_2_^-^ formed was calculated using a standard calibration curve prepared by NaNO_2_ and was expressed in terms of μmol NO_2_^-^ formed g^-1^ FW h^-1^.

The assay of nitrite reductase (NiR; EC 1.7.7.1) activity is based on the reduction in the content of nitrite in the reaction mixture as described by Debouba et al. (2006). The absorbance of reaction mixture was recorded at 540 nm and NiR activity was expressed in terms of μmol NO_2_^-^ reduced g^-1^ FW h^-1^.

### Estimation of Ammonium Assimilating Enzymes

#### GS-GOGAT Pathway

##### Glutamine synthetase (GS) and glutamate synthase (GOGAT) activities

Glutamine synthetase (GS; EC 6.3.1.2) activity was assayed by the method of Lillo (1984). The absorbance of the reaction mixture was monitored spectrophotometrically at 540 nm and the enzyme activity was expressed in terms of A_540__nm_ g^-1^ FW h^-1^.

Glutamine-2-oxoglutarate aminotransferase or glutamate synthase (NADH-GOGAT; EC 1.4.1.14) activity was assayed following the method of Singh and Srivastava (1986). The decrease in absorbance of the reaction mixture was recorded spectrophotometrically at 340 nm for 5 min and the activity of GOGAT was calculated by standard curve prepared with NADH and expressed in terms of nmol NADH oxidized g^-1^ FW h^-1^.

#### Alternate (NADH-GDH) Pathway

##### Glutamate dehydrogenase (GDH) activity

The aminating activity of glutamate dehydrogenase (GDH; EC 1.4.1.2) was assayed following the method of Singh and Srivastava (1983). The reaction was initiated by adding the substrate ammonium sulfate in the reaction mixture and decrease in absorbance was read at 340 nm for 5 min. The activity of GDH enzyme was calculated by the standard curve prepared with NADH and expressed in terms of nmol NADH oxidized g^-1^ FW h^-1^.

### Estimation of Contents of Protein and Carbohydrate

The contents of protein and carbohydrate in root and leaf tissues were measured spectrophotometrically at 595 and 490 nm, following the procedure of Bradford (1976) and Dubois et al. (1956), respectively.

### Statistical Analysis

Results were statistically analyzed by analysis of variance (ANOVA). Tukey test was applied for the mean separation for significant differences among treatments at *P* < 0.05 levels. The results presented are the means of three replicates (*n* = 3) to check the reproducibility of the results.

## Results

### Growth Attributes

**FIGURE 1:**
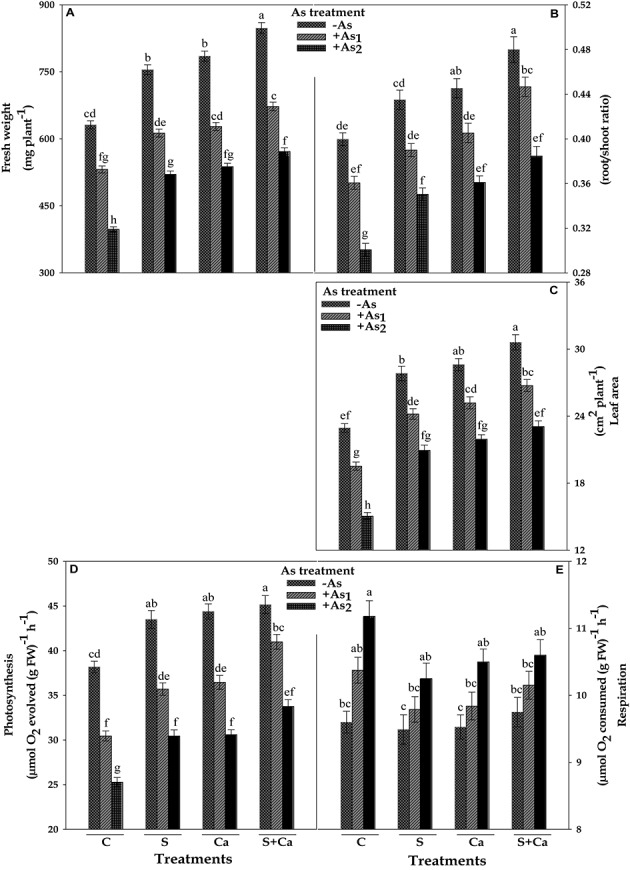
Impact of exogenous application of S and Ca individually and in combination on fresh weight **(A)**, root/shoot ratio **(B)**, leaf area **(C)**, photosynthetic O_2_ evolution **(D)**, and respiratory rate **(E)** of *Brassica juncea* L. seedlings exposed to As_1_ (15 mg arsenic kg^-1^ sand) and As_2_ (30 mg arsenic kg^-1^ sand) stress. Data represent the mean value ± standard error of three replicates (*n* = 3). Bars followed by different letters show significant difference according to Tukey test at *P* < 0.05 significance level.

The impact of S, Ca, and S+Ca on As stressed *Brassica* seedlings was examined by analyzing the FW of the plant, root/shoot ratio, and leaf area, which have been portrayed in **Figure 1**. Both the doses of As, i.e., As_1_ and As_2_, significantly (*P* < 0.05) declined the FW by 16 and 37%, root/shoot ratio by 9 and 24%, and leaf area by 15 and 34%, respectively, over the values of control. Contrastingly, exogenous application of S and Ca alone and in combination (S+Ca) markedly ameliorated the negative effect of As on above parameters. The S+Ca application completely recovered the negative effect of As under As_1_ stress on FW and root/shoot ratio; while only 9 and 4% inhibition, respectively, under As_2_ stress in comparison to As_2_ stress alone was observed except for leaf area where the negative effect of both the doses i.e., As_1_ and As_2_ was totally recovered.

### Metal Accumulation

The results pertaining to As, S, and Ca accumulation were measured in terms of As/S and As/Ca ratio in root and shoot (stem+leaves) and have been portrayed in **Table 1**. Arsenic at both the doses, i.e., As_1_ and As_2_, sharply enhanced the ratio of As/S from 0.080 to 0.146 and As/Ca from 0.103 to 0.195 in roots, respectively, over the values of control. Corresponding increase in the ratio of As/S in shoots was from 0.053 to 0.081 and in case of As/Ca, it was enhanced from 0.048 to 0.076. However, upon addition of S and Ca either alone or in combination to As stressed *Brassica* seedlings, a significant reduction in As/S and As/Ca ratio was noticed and the maximum reduction was found under S+Ca treatment.

**Table 1:** Impact of exogenous application of S and Ca individually and in combination on photosynthetic pigment contents in leaves and As/S and As/Ca ratio in root and shoot of *Brassica juncea* L. seedlings exposed to As_1_ (15 mg arsenic kg^-1^ sand) and As_2_ (30 mg arsenic kg^-1^ sand) stress.

Treatments	Total Chl (*a*+*b*)	Photosynthetic pigments		As/S ratio		As/Ca ratio	
	(mg g^-1^ FW)	Chl *a*/*b*	Total Chl/Car	Root	Shoot	Root	Shoot
Control	1.779 ± 0.028^bc^	2.940 ± 0.051^bc^	5.129 ± 0.086^b^	Nd	nd	nd	nd
As_1_	1.621 ± 0.066^cd^	3.218 ± 0.057^ab^	4.940 ± 0.092^ab^	0.0800 ± 0.0014^b^	0.0532 ± 0.0009^b^	0.1029 ± 0.0019^b^	0.0484 ± 0.0008^b^
As_2_	1.479 ± 0.027^e^	3.299 ± 0.069^a^	4.867 ± 0.101^b^	0.1458 ± 0.0030^a^	0.0814 ± 0.0016^a^	0.1947 ± 0.0040^a^	0.0765 ± 0.0015^a^
S	1.841 ± 0.040^a^	2.917 ± 0.066^bc^	5.174 ± 0.118^bc^	Nd	nd	nd	nd
As_1_+S	1.751 ± 0.035^cd^	2.969 ± 0.057^bc^	5.095 ± 0.109^bc^	0.0147 ± 0.0002^e^	0.0082 ± 0.0001^cd^	0.0204 ± 0.0004^e^	0.0087 ± 0.0001^cd^
As_2_+S	1.579 ± 0.034^de^	3.132 ± 0.069^ab^	4.900 ± 0.101^c^	0.0346 ± 0.0007^d^	0.0106 ± 0.0002^c^	0.0450 ± 0.0010^cd^	0.0105 ± 0.0002^c^
Ca	1.866 ± 0.037^ab^	2.904 ± 0.054^c^	5.205 ± 0.101^b^	Nd	nd	nd	nd
As_1_+Ca	1.781 ± 0.035^bc^	2.913 ± 0.060^bc^	5.148 ± 0.109^bc^	0.0151 ± 0.0003^e^	0.0080 ± 0.0001^cd^	0.0182 ± 0.0003^e^	0.0076 ± 0.0001^de^
As_2_+Ca	1.602 ± 0.028^de^	3.095 ± 0.055^ab^	4.929 ± 0.086^c^	0.0330 ± 0.0005^d^	0.0104 ± 0.0001^c^	0.0395 ± 0.0007^d^	0.0094 ± 0.0001^cd^
Ca+S	1.964 ± 0.043^a^	2.891 ± 0.063^c^	5.321 ± 0.121^b^	Nd	nd	nd	nd
As_1_+Ca+S	1.846 ± 0.037^ab^	2.874 ± 0.057^c^	5.262 ± 0.107^b^	0.0178 ± 0.0003^e^	0.0057 ± 0.0001^d^	0.0226 ± 0.0004^e^	0.0054 ± 0.0001^e^
As_2_+Ca+S	1.641 ± 0.035^cd^	3.054 ± 0.066^ab^	4.947 ± 0.106^b^	0.0404 ± 0.0008^c^	0.0066 ± 0.0001^d^	0.0481 ± 0.0010^c^	0.0055 ± 0.0001^e^

*Data represent the mean value ± standard error of three replicates (n = 3). Values within same column followed by different letters show significant difference at P < 0.05 significance level.*

### Photosynthetic Pigments

The results pertaining to photosynthetic pigments content were measured in terms of total chlorophyll (Chl *a*+*b*) content and the ratios of Chl *a*/*b* and total Chl/Car which have been depicted in **Table 1**. Results revealed that both the doses of As, i.e., As_1_ and As_2_ caused significant (*P* < 0.05) damage showing a decrease of 9 and 17% in total Chl content and 4 and 5% in Chl/Car ratio; while a significant increase of 9 and 12% in Chl *a*/*b* ratio, respectively, over the value of respective control was noticed. Exogenous supplementation of Ca and S+Ca completely recovered the loss to Chl content and also Chl/Car ratio in As_1_ stressed seedlings while with S supplementation still a marginal decrease was noticed. Under As_2_ stress though the supplementation of S, Ca, and S+Ca resulted into significant improvement in the pigments content, the seedlings still showed a decrease of 11, 10, and 8% in total Chl content and 4, 4, and 3% in Chl/Car ratio, respectively. Furthermore, the increased value of Chl *a*/*b* due to As_1_ and As_2_ treatment decreased significantly with the supplementation of S, Ca, and S+Ca and this effect was more prominent with combined treatment of S and Ca.

### Photosynthesis and Respiration

The results pertaining to photosynthetic oxygen evolution and respiratory oxygen uptake have been framed in **Figure 1**. The results showed that As_1_ and As_2_ doses declined the photosynthetic oxygen evolution rate by 20 and 34%; contrastingly, the respiratory oxygen uptake rate was enhanced by 8 and 16%, respectively, over the values of respective control. However, exogenous application of S, Ca, and S+Ca significantly ameliorated the damaging effect on photosynthetic oxygen evolution rate with more pronounced effect of S+Ca treatment under As_1_ stress which showed a complete amelioration, while only 12% inhibition was noticed under As_2_ treatment. Contrastingly, under As stress, S, Ca, and S+Ca supplementation resulted into significant decrease in respiratory rate; however, the values were still higher than that of untreated control.

### Chlorophyll *a* Fluorescence Transient

**FIGURE 2:**
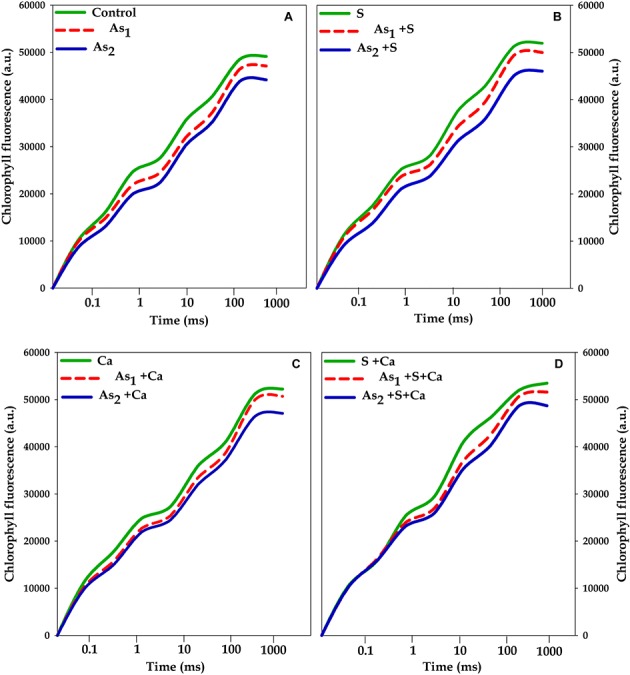
Impact of exogenous application of S **(B)** and Ca **(C)** individually and in combination **(D)** on OJIP transient of *Brassica juncea* L. seedlings exposed to As_1_ (15 mg arsenic kg^-1^ sand) and As_2_ (30 mg arsenic kg^-1^ sand) stress **(A)**.

**FIGURE 3:**
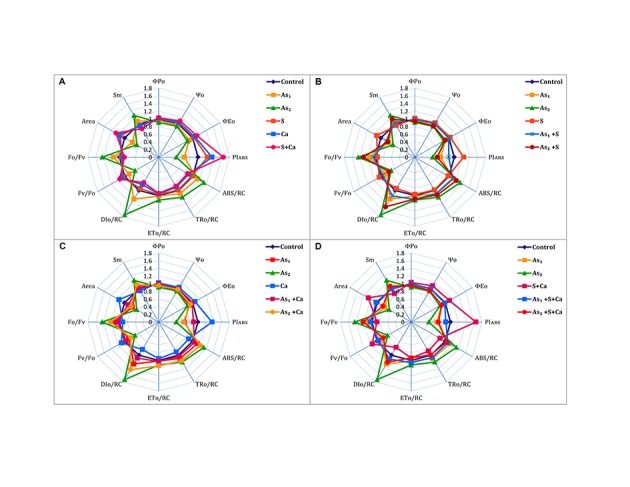
Impact of exogenous application of S **(B)** and Ca **(C)** individually and in combination **(D)** on chlorophyll fluorescence indices of *Brassica juncea* L. seedlings exposed to As_1_ (15 mg arsenic kg^-1^ sand) and As_2_ (30 mg arsenic kg^-1^ sand) stress **(A)**.

The effects of tested doses of As on Chl *a* fluorescence characteristics in *Brassica* seedlings exposed to S, Ca, and S+Ca were measured by OJIP test and the results have been portrayed in **Figures 2**, **3**. A characteristic OJIP transient rise was noticed. While a sharp decrease in O–J, J–I, and I–P rise was recorded under As_1_ and As_2_ treatments (**Figure 2**). Upon exogenous application of S, Ca, and S+Ca either alone or to As treated *Brassica* seedlings, a significant amelioration in decrease of O–J, J–I, and I–P rise was observed.

The ΦP_0_, Ψ_0_, ΦE_0_, PI_ABS_, and Area of PS II sharply declined under As stress in dose dependent manner over the control values (**Figure 3**). On the other hand, exogenous application of S, Ca, and S+Ca to As treated test seedlings significantly alleviated the damaging effect and a marginal increase in ΦP_0_, Ψ_0_, ΦE_0_, PI_ABS_, and Area was recorded. Arsenic treatment also decreased the size and number of active reaction centers (F_v_/F_0_) in the photosynthetic apparatus that was symbolized by increased energy fluxes parameters, i.e., ABS/RC, TR_0_/RC, DI_0_/RC, and ET_0_/RC along with the increase in F_0_/F_v_ and *S*_m_ values. However, S, Ca, and S+Ca significantly improved the proportion of size and number of active reaction centers thereby declining the values for energy flux parameters and F_0_/F_v_ and *S*_m_ when subjected to As stressed seedlings.

### Inorganic Nitrogen Contents

**FIGURE 4:**
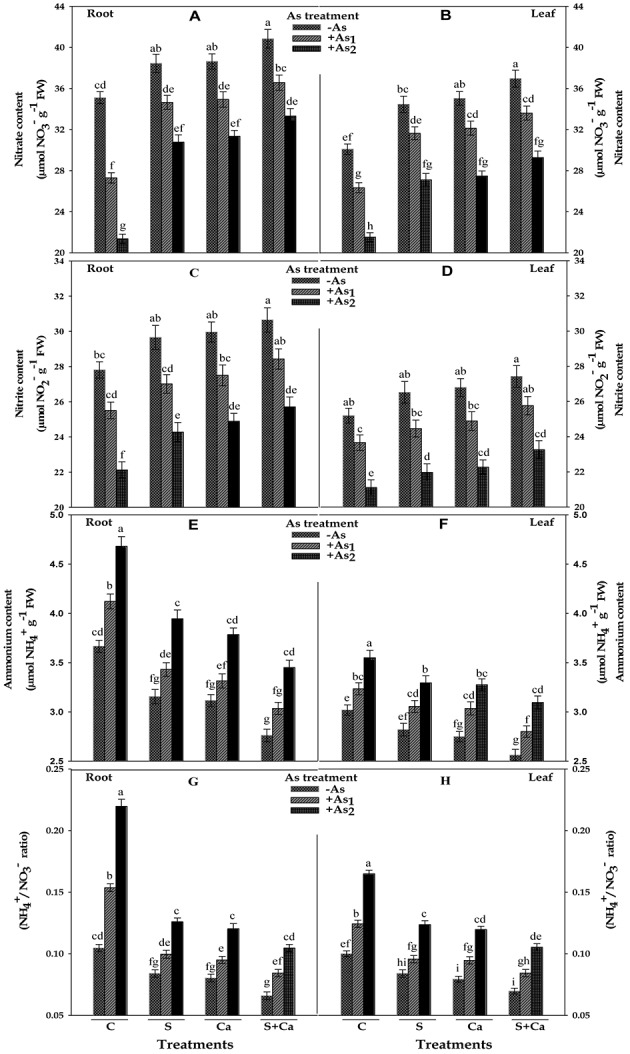
Impact of exogenous application of S and Ca individually and in combination on inorganic nitrogen contents: nitrate **(A,B)**, nitrite **(C,D)**, and ammonium **(E,F)** and NH_4_^+^/NO_3_^-^ ratio **(G,H)** in root and leaves of *Brassica juncea* L. seedlings exposed to As_1_ (15 mg arsenic kg^-1^ sand) and As_2_ (30 mg arsenic kg^-1^ sand) stress. Data represent the mean value ± standard error of three replicates (*n* = 3). Bars followed by different letters show significant difference according to Tukey test at *P* < 0.05 significance level.

Results pertaining to inorganic nitrogen contents, i.e., NO_3_^-^, NO_2_^-^, and NH_4_^+^ and NH_4_^+^/NO_3_^-^ ratio in test seedlings have been portrayed in **Figure 4**. The results suggested that As_1_ and As_2_ doses markedly reduced the contents of NO_3_^-^ by 22 and 39%, NO_2_^-^ by 8 and 20% in roots and corresponding decrease in the contents of NO_3_^-^ it was 12 and 28% and of NO_2_^-^ it was 6 and 16% of NO_2_^-^ it was in leaves, respectively, with respect to control. Contrastingly, NH_4_^+^ content was increased by 12 and 28% in root and 7 and 18% in leaves, respectively, under As_1_ and As_2_ stress; therefore, a sharp increase in NH_4_^+^/NO_3_^-^ ratio in both root and leaves was noticed. When As_1_ and As_2_ stressed seedlings were subjected to S, Ca, and S+Ca treatment, the levels of NO_3_^-^ and NO_2_^-^ were found to improve in both root and leaves and under S+Ca treatment the levels of NO_3_^-^ and NO_2_^-^ in As_1_ treated seedlings were found to be even more than the level of control sample (**Figure 4**). Reverse to this, As induced increment in NH_4_^+^ accumulation in root and leaves exhibited a declining trend following supplementation with S, Ca, and S+Ca; hence, a substantial decrease in NH_4_^+^/NO_3_^-^ ratio was noticed under individual as well as combined supplementation with S and Ca even in As stressed seedlings.

### Activities of Nitrate Assimilating Enzymes

**FIGURE 5:**
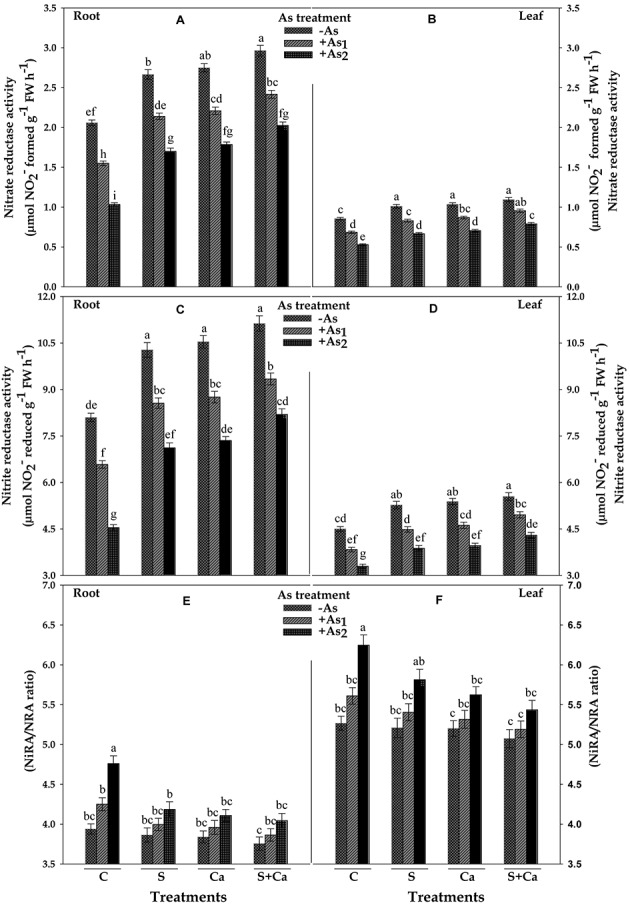
Impact of exogenous application of S and Ca individually and in combination on activities of nitrate assimilating enzymes: nitrate reductase (NRA) **(A,B)** and nitrite reductase (NiRA) **(C,D)** and NiRA/NRA ratio **(E,F)** in root and leaves of *Brassica juncea* L. seedlings exposed to As_1_ (15 mg arsenic kg^-1^ sand) and As_2_ (30 mg arsenic kg^-1^ sand) stress. Data represent the mean value ± standard error of three replicates (*n* = 3). Bars followed by different letters show significant difference according to Tukey test at *P* < 0.05 significance level.

The results related with activities of NR (NRA) and NiR (NiRA) and the ratio of NiRA/NRA in test seedlings have been portrayed in **Figure 5**. Under As_1_ and As_2_ stress, the activity of NR was decreased by 25 and 50% and NiR by 19 and 44% in root and corresponding decrease in the activities of NR was 20 and 38% and in NiR it was 15 and 27% in leaves, respectively, over their control values. Furthermore, NiRA/NRA ratio to root as well as in leaves was found to increase with As treatment which was maximum under As_2_ stress. Upon S, Ca, and S+Ca supplementation, the negative effect on NR and NiR activities caused by As_1_ and As_2_ stress was alleviated; thus, a significant decline in NiRA/NRA ratio in both root and leaves was observed (**Figures 5A–F**). Moreover, S+Ca treatment to As stressed seedlings was found to be more effective in alleviating the negative effect on NR and NiR activities.

### Activities of Ammonium Assimilating Enzymes

**FIGURE 6:**
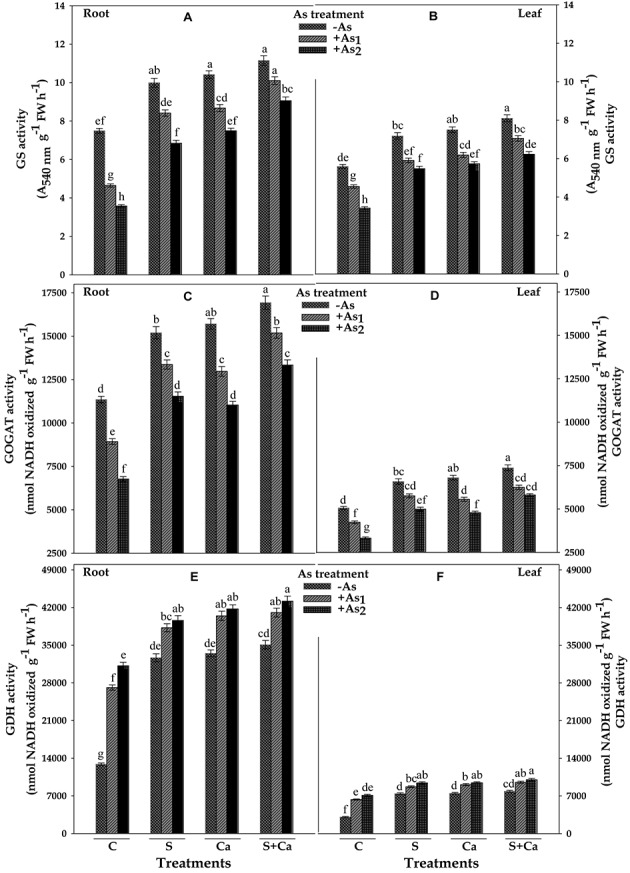
Impact of exogenous application of S and Ca individually and in combination on activities of ammonium assimilating enzymes: glutamine synthetase (GS) **(A,B)**, glutamate synthase (GOGAT) **(C,D)**, and glutamate dehydrogenase (GDH) **(E,F)** in root and leaves of *Brassica juncea* L. seedlings exposed to As_1_ (15 mg arsenic kg^-1^ sand) and As_2_ (30 mg arsenic kg^-1^ sand) stress. Data represent the mean value ± standard error of three replicates (*n* = 3). Bars followed by different letters show significant difference according to Tukey test at *P* < 0.05 significance level.

The results showing the effect of As on activities of ammonium assimilating enzymes, i.e., GS, GOGAT, and GDH of test plant have been depicted in **Figure 6**. The results revealed that As_1_ and As_2_ treatment decreased the GS activity by 38 and 52% and GOGAT by 21 and 40% in root and corresponding decrease in the GS activity was 19 and 39% and in GOGAT it was 16 and 34% in leaves, respectively, as compared to control. When As_1_ and As_2_ treated seedlings were supplied with S, Ca, and S+Ca, significant improvement in the GS and GOGAT activities of root and leaves of test plant was noticed and was maximum under S+Ca supplementation. In contrast to this, GDH activity under As stress exhibited appreciable enhancement showing a rise of 110 and 142% in root and 106 and 131% in leaves and the activity was further accelerated upon S+Ca application as it was raised by 217 and 235% in root and 209 and 224% in leaves in As_1_ and As_2_ stressed test seedlings, respectively, over the values of control.

### Protein and Carbohydrate Contents

**FIGURE 7:**
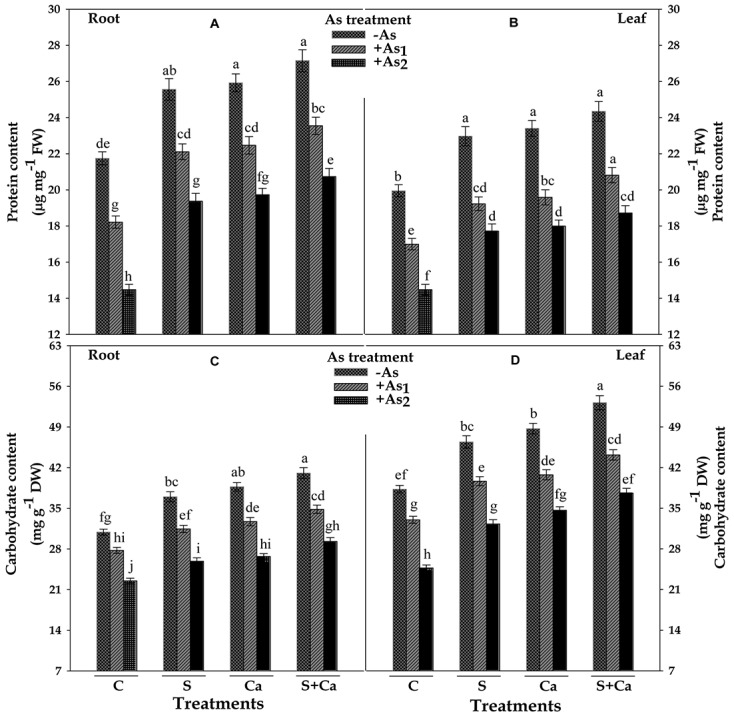
Impact of exogenous application of S and Ca individually and in combination on protein **(A,B)** and carbohydrate **(C,D)** contents in root and leaves of *Brassica juncea* L. seedlings exposed to As_1_ (15 mg arsenic kg^-1^ sand) and As_2_ (30 mg arsenic kg^-1^ sand) stress. Data represent the mean value ± standard error of three replicates (*n* = 3). Bars followed by different letters show significant difference according to Tukey test at *P* < 0.05 significance level.

Results pertaining to protein and carbohydrate contents in root and leaves of test seedlings have been portrayed in **Figure 7**. The results revealed that As_1_ and As_2_ doses of As, noticeably declined the content of protein by 16 and 33%, carbohydrate by 10 and 27% in root, and the corresponding decline in the content of protein was 15 and 27% and of carbohydrate it was 14 and 35% in leaves, respectively, in respect of control. However, exogenous application of S, Ca, and S+Ca to As_1_ and As_2_ stressed seedlings significantly ameliorated the negative effect of As on protein and carbohydrate contents and the effect was more pronounced in case of root as well as leaves under As_1_ stress (**Figure 7**).

**Table 2** shows results for two-way ANOVA test for arsenic and mineral nutrients (S and Ca) on the studied parameters. Both the variables when subjected individually to the seedlings significantly affected all the studied parameters (except for the ratio for root to shoot). However, the interactive effect of both the variables was significant on FW, root/shoot, nitrate content, NH_4_^+^/NO_3_^-^, NiRA/NRA, and GS and GDH activities and due to their significant impact on nitrate content and important enzymes of ammonium assimilation, better growth was noticed in comparison to S and Ca alone.

**Table 2:** Two-way ANOVA results for individual and interactive effect of arsenic and nutrients on the studied parameters in *Brassica* L. seedlings.

Factor	[As]	[Nutrient]	[As X nutrient]	Factor	[As]	[Nutrient]	[As X nutrient]
FW of plant				NH_4_^+^/NO_3_^-^ ratio			
df	2	3	6	df	2	3	6
*F*	751.00	194.39	4.18	*F*	397.21	365.63	40.01
*P*-value	**<0.001**	**<0.001**	**<0.001**	*P*-value	**<0.001**	**<0.001**	**<0.001**
Root/shoot ratio				NR activity			
df	2	3	6	df	2	3	6
*F*	1.53	0.76	1.00	*F*	475.65	234.33	0.36
*P*-value	0.235	0.526	**<0.001**	*P*-value	**<0.001**	**<0.001**	0.891
Leaf area				NiR activity			
df	2	3	6	df	2	3	6
*F*	209.53	130.75	0.46	*F*	329.19	178.55	1.19
*P*-value	**<0.001**	**<0.001**	0.827	*P*-value	**<0.001**	**<0.001**	0.345
Photosynthesis				NiRA/NRA ratio			
df	2	3	6	df	2	3	6
*F*	286.81	68.75	1.54	*F*	26.51	15.13	2.58
*P*-value	**0.041**	**<0.001**	0.208	*P*-value	**<0.001**	**<0.001**	**<0.001**
Nitrate content				GS activity			
df	2	3	6	df	2	3	6
F	167.73	94.33	5.14	*F*	300.97	419.38	6.48
*P*-value	**<0.001**	**<0.001**	**<0.001**	*P*-value	**<0.001**	**<0.001**	**<0.001**
Nitrite content				GOGAT content			
df	2	3	6	df	2	3	6
*F*	90.34	16.96	0.14	*F*	238.65	263.69	1.74
*P*-value	**<0.001**	**<0.001**	0.988	*P*-value	**<0.001**	**<0.001**	0.155
Ammonium content df	2	3	6	GDH activity df	2	3	6
*F*	120.33	114.70	1.27	*F*	219.06	302.15	14.21
*P*-value	**<0.001**	**<0.001**	0.306	*P*-value	**<0.001**	**<0.001**	**<0.001**

*The bold values show significant level (P-values) for arsenic and nutrients.*

## Discussion

The present study was designed to explicate the S and Ca induced recovery of C and N skeleton in terms of photosynthesis and nitrogen metabolism in As-challenged *Brassica* seedlings. From the results, it is clear that As exhibited toxicity to *Brassica* at both the doses by posing negative effect on growth attributes measured in terms of FW, root/shoot ratio, and leaf area (**Figures 1A–C**). The As induced reduction in root/shoot ratio could be a resultant of higher As accumulation in root as it is in direct contact of sand that severely damaged the roots thereby resulting into lower root/shoot ratio (**Table 1**). Further, the reduction in leaf area could be attributed to: (i) decrease in light harvesting components (**Table 1**) and consequently the photosynthetic rate (**Figure 1**), (ii) As induced overproduction of ROS (Singh et al., 2018), and (iii) decreased activities of nitrogen assimilating enzymes (**Figures 5**, **6**). Arsenic accumulation in *Brassica* seedlings showed higher As/S and As/Ca ratio in root than shoots, indicating that greater amount of As was restricted in roots and less was translocated to shoot (**Table 1**). The higher As accumulation in roots could also be due to the damage and alteration in plasma membrane and cell wall structures as was suggested by Singh et al. (2013). On the other hand, application of S and Ca either alone or in combination regulated the As toxicity thereby improving the root/shoot ratio and leaf area in test plant, which probably could be due to: (i) upregulation in the uptake and translocation of water and mineral nutrients [Mn, K, Mg, Fe, Cu, Zn, and P including S and Ca (data not shown)], which are necessary as cofactors in different enzymes, chlorophyll biosynthesis, photosynthetic activity (Sebastian and Prasad, 2014), etc., (ii) by recovering membrane integrity that was lost due to As accumulation (Ahmad et al., 2015) due to which higher As/S and As/Ca ratio in roots was observed (**Table 1**), (iii) by favoring the Ca mediated cell elongation and expansion (Ahmad et al., 2016), and (iv) by improving the activities of nitrogen and ammonium assimilating enzymes (**Figures 5**, **6**). When As stressed seedlings were treated with exogenous S, Ca, and S+Ca, a decrease in As/S and As/Ca ratio was observed in both root and shoot, which was more pronounced in roots than shoot thereby signifying that more S and Ca were maintained in roots that might have played an important role in the formation of thiol containing compounds and in free radical scavenging (Price et al., 1994; Dixit et al., 2015) to detoxify greater amount of As and ROS induced by As in roots.

**FIGURE 8:**
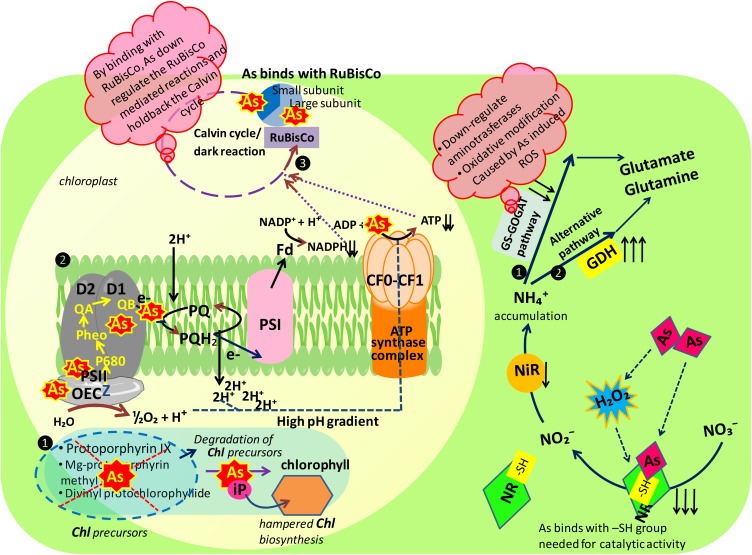
Schematic representation of major mechanisms underlying arsenic toxicity in *Brassica juncea* L. seedlings. Figure illustrating the mode of action of arsenic at probable sites of two major mechanisms playing major role in plant metabolism: photosynthesis and nitrogen metabolism, As, arsenic; inorganic nitrogen contents: NO_3_^-^, nitrate; NO_2_^-^, nitrite, and NH_4_^+^, ammonium; nitrate assimilating enzymes: NR, nitrate reductase and NiR, nitrite reductase; ammonia assimilating enzymes: 1. GS-GOGAT pathway: GS, glutamine synthetase and GOGAT, glutamine 2-oxoglutarate aminotransferase; 2. alternative pathway: GDH, glutamate dehydrogenase; photosynthetic apparatus: Chl, chlorophyll; OEC, oxygen evolving complex; PS I, photosystem I; PS II, photosystem II; Fd, ferredoxin; PQ, plastoquinone; -SH, sulfhydryl group.

Being essential components of photosynthesis, photosynthetic pigments (total chlorophyll, i.e., Chl *a*+*b*; Chl *a*/*b* and Chl/Car) were also analyzed and found to be severely damaged when exposed to As stress in dose-dependent manner (**Table 1** and **Figure 8**). This As induced damaging effect on photosynthetic pigments could be due to: (i) the competition as well as replacement of inorganic phosphate (iP), needed for Chl biosynthesis (Mishra et al., 2016), (ii) repression of the activities of enzymes (require –SH) needed for pigment biosynthesis (Singh et al., 2013), (iii) degradation of chlorophyll biosynthesis precursors (Mishra et al., 2016), and (iv) oxidation of photochemical apparatus that damages photosynthetic pigments (Stoeva et al., 2005). Our findings show conformity with the studies of Singh et al. (2016) and Mishra et al. (2016) in As-challenged *Oryza sativa* and *Ceratophyllum demersum*, respectively. Arsenic also leads to chlorosis by upregulating the conversion of Chl *b* into Chl *a*; hence, relatively higher loss in Chl *b* content causes higher Chl *a*/*b* ratio, which is the symptom of inadequacy of peripheral antenna system. Contrastingly, Chl/Car ratio was decreased with increasing concentration of As (**Table 1**) thereby implying a great loss in Car content that might have occurred due to the damage to thylakoid membrane, which is in consonance with the findings of Stoeva et al. (2005). However, exogenous S and Ca improved the Chl biosynthesis by improving the uptake of Mn^2+^, Zn^2+^, Fe^2+^, and Mg^2+^ (data not shown) and also improving the Car content. Calcium induced improvement in Chl synthesis could be due to its role as secondary messenger in cytokinin action as cytokinin is involved in distribution of mineral nutrients within plants (Lechowski and Białczyk, 1993).

Any alteration in chlorophyll biosynthesis directly hinders the process of photosynthesis, and therefore affects plant growth. In our study, As adversely affects photosynthetic O_2_ yield and the extent of inhibition was concentration dependent (**Figure 1**). The possible reason behind the loss of photosynthetic efficiency could be: (i) damage to the photosynthetic pigments (**Table 1**) and membranes harboring the photosystems (Rahman et al., 2007), (ii) Chl is used as a source of metabolic carbon when carbohydrate availability is low (Araújo et al., 2011), (iii) damage to PS II reaction centers (**Figure 3**) along with the reduction in photosynthetic linear electron flow (LEF; **Figure 2**) thereby decreasing the potential to produce ATP and NADPH, both of which are needed to fuel the carbon fixation reactions (**Figure 8**), and (iv) the significant decline in 29 kDa ribonucleoproteins thereby holding back the dark reactions/Calvin cycle of photosynthesis. Moreover, As (As^V^) most possibly has replaced iP in photophosphorylation reactions thereby uncoupling electron transport for ATP synthesis in thylakoid (Watling-Payne and Selwyn, 1974). Interestingly, both S and Ca supplementation either alone or in combination to As stressed *Brassica* plant noticeably improved the photosynthetic O_2_ evolution rate (**Figure 1**). The increase in photosynthetic rate could be explained on the basis that As stressed plant requires more energy to maintain the cellular homeostasis, so, in order to fulfill the energy demand, exogenous S might have up regulated the ferredoxin-NADP reductase (FNR) level, that catalyzes the production of NADPH+H^+^ required for CO_2_ assimilation and energy production (Dixit et al., 2015). High S concentration may also up regulate the Fe–S clusters, which is essential and versatile cofactors of proteins involved in catalysis and electron transport (Lill, 2009). The increased photosynthetic efficiency could also be drawn on the basis that S and Ca might have partially replaced the LEF thereby switching on the cyclic electron flow (CEF) around PSI, where CEF can oxidize P_700_ into P_700_^+^ and consumes excess reducing power of NADPH through the NADPH dehydrogenase-dependent pathway, which provides energy to Calvin cycle (Hochmal et al., 2015) thereby meeting energy demand. Most possibly exogenous Ca stores by binding to the proteins or the thylakoid membrane and regulates CEF via calcium sensor proteins (CAS; Hochmal et al., 2015).

To pinpoint the targets/sites of As on photosynthesis, polyphasic Chl *a* fluorescence transient was analyzed (**Figure 2**). The OJIP transient signifies sequential reduction of electron transport pool of PS II (Govindjee, 1995; Misra et al., 2012; Kalaji et al., 2014, 2017). In the present study, the intensity of fluorescence in the OJIP transient decreased with increasing concentration of As (**Figure 2**), which may be due to: (i) inhibition of electron transport at PS II donor side, thereby resulting into P_680_^+^ accumulation (Govindjee, 1995) and (ii) due to decreased pool size of Q_A_^-^ (Kalaji et al., 2014). The kinetic parameters for Chl *a* fluorescence, i.e., Ψ_0_, ΦP_0_, ΦE_0_, as well as PI_ABS_, were found to diminish after As treatment (**Figure 3**) that suggests toward the sluggish electron flow from PS II to PS I and limitation of Q_A_^-^ reoxidation which could be associated with poor diffusion of PQ across the thylakoid membranes (Magyar et al., 2018). The increase in S_m_ value under As stress implied that the heterogeneity of PQ increased the electron donation capacity and Q_A_ reduction on acceptor side of PS II, which indicates that As decreased the total electron accepting capacity. The adverse effect of As on performance of PS II photochemistry might have occurred due to: (i) As accumulation inside the chloroplast that might have interrupted the functioning of thylakoid membranes, (ii) significant decrease in number of active RCs as clear from F_v_/F_0_ values, thereby increasing the energy flux parameters, i.e., ABS/RC, TR_0_/RC, ET_0_/RC, and DI_0_/RC of PS II, (iii) downstream of Q_A_^-^ electron transfer thereby changing the rate of electron flow from Q_A_^-^ to Q_B_, (iv) by increasing the degradation rate of D1 protein of PS II that shifted the equilibrium between synthesis and degradation thereby leading into inactivation of RCs, and (v) impairment of PS II on donor side (Tóth et al., 2012). The decline in the size and number of active photosynthetic reaction centers (F_v_/F_0_) indicates that As either decreased the rate of photochemistry or pool size of Q_A_ (Krause and Weiss, 1991). The lowered PI_ABS_ of As stressed test plant was due to damaging effects on traits of PSII photochemistry: ΦP_0_, Ψ_0_, and ΦE_0_. Furthermore, increased F_0_/F_v_ value under As treatments indicated toward As-induced damaging effects on OEC, which could be due to decline in uptake of nutrients like Mn, which is an important component of OEC (data not shown). The As induced reduction in Area (symbolizes pool size of Q_A_ on the PS II reduction side) under As stress indicates that As inhibited electron transport rate at PS II donor side. Results clearly revealed that As affected both the acceptor as well as the donor side of PS II, thereby impairing the photosynthetic performance (**Figure 1**). On the other hand, S and Ca alone and in combination restored the functional and structural attributes of PS II such as quantum yield, size and number of RCs, and OEC as evidence by increased ΦP_0_ and F_v_/F_0_ values and decreased F_0_/F_v_ values, respectively. On S and Ca application to As stressed *Brassica* seedlings, a steep decline in ABS/RC, TR_0_/RC, ET_0_/RC, and DI_0_/RC (**Figure 3**) was obtained indicating that PS II apparatus maintained the equilibrium of energy fluxes for absorption, trapping, and electron transport through regulation of active RCs of PS II in response to As stress (Misra et al., 2001). This might have occurred due to higher plastoquinone levels, P_700_-content, and Hill activity as reported by Buschmann and Lichtenthaler (1977) following S and Ca treatments. Indeed, in the present study, the improved Area value (proportional to the pool size of the electron acceptors Q_A_ on the reduction side of PS II) under S and Ca application particularly S+Ca indicates that it might be an outcome of increase in the electron transfer from the reaction centers to the quinone pool. In addition to this, S and Ca might have recovered D1 protein either by increasing its synthesis or by reducing the degradation rate that finally improved the efficiency of PS II.

The dark respiratory O_2_ uptake rate was increased with increasing doses of As (**Figure 1**), which could be the result of partial uncoupling of ETC due to thylakoid membrane disruption; therefore, increase in O_2_ uptake rate was recorded to meet the situation of both, i.e., disrupted respiratory apparatus and the demand of ATP for carrying out the basic life processes by increasing the respiratory electron transport rate (Prasad and Zeeshan, 2005). Another possibility could be excessive ROS production that possibly consumes more O_2_ (actively accepts electron at intermediate stage of respiratory and photosynthetic ETC); therefore, an obvious demand of O_2_ was observed. The marginal decrease in the rate of dark respiration under S and Ca treatment could be due to the proper functioning of basal metabolism as S involves in various cellular redox processes and also increased the S assimilation by enhancing ATPS activity (Liang et al., 2016).

Nitrogen metabolism is an important biochemical process which maintains the N-status of plants; therefore, different indices were studied to get deeper insight regarding the degree of damage posed by As stress, while protein synthesis is closely linked with the formation of new tissues, which are major sink for N compounds. In plants, NO_3_^-^ the most preferential source of nitrogen once absorbed by roots is assimilated to NH_4_^+^ through sequential reduction by NR and NiR. In the present study, NO_3_^-^ content was severely affected by As in dose-dependent manner (**Figure 4**) which could be due to: (i) overproduction of ROS that causes membrane damage and electrolyte leakage, which might decreased NO_3_^-^ absorption by root cells, (ii) uptake competition between NO_3_^-^ and other existing anions like Cl^-^, SO_4_^2-^, and CO_3_^2-^, which may have unfavorable influences on plasma membrane permeability, and (iii) alteration in transpiration rate, which may result into limited NO_3_^-^ flux from root to leaves. Being a committed enzyme in NO_3_^-^ assimilation, NR is very sensitive to As stress; therefore, a marked inhibition in NR activity under As (**Figure 5**) stress was recorded that may restrict NO_2_^-^ formation in comparison to control. It is known that –SH groups are required for catalytic activity of NR (**Figure 8**; Solomonson and Barber, 1990), so it is possible that As might have interacted with this group present in the active sites of NR enzyme (Hartley-Whitaker et al., 2002) thereby hampering it. Another possibility could be that As induces accumulation of endogenous H_2_O_2_, and NR is very sensitive to H_2_O_2_ as reported by Sharma and Dubey (2005), so H_2_O_2_ might have reduced the activity of this enzyme. Both NR and NiR enzymes are co-regulated on the induction side (Migge et al., 1997). Similar to NR, NiR activity was also inhibited by As (**Figure 5**) but less affected than NR as have been reported in *Pteris vittata* and *Pteris ensiformis* (Singh et al., 2009). The possible explanation could be, as the main regulator of NiR activity is the activity of NR, which is induced by NO_3_^-^ (substrate; Dguimi et al., 2009), so the decline in NiR activity may be associated with reduced availability of NO_2_^-^ ions, which is considered to be originated primarily from NR-catalyzed NO_3_^-^ reduction. Another possibility may be heavy damage to photosynthetic ETC, which donates electron to reduce NO_2_^-^ via ferredoxin. Further, As may decrease NR and NiR activities by affecting the enzyme synthesis or by bringing conformational changes in the enzymes’ structure. On the other hand, S and Ca supplementation either alone or in combination to As stressed seedlings enhanced NO_3_^-^ content in tissues (**Figure 4**), which might be due to increased rate of transpiration (Wu et al., 2011) that facilitated the absorption and translocation of NO_3_^-^ to leaves; as a consequence, NR and NiR activities were increased. Furthermore, comparatively less effect on NR activity under S and Ca treatment could be correlated with the betterment in NO_3_^-^ content under S and Ca, and NR is a substrate-inducible enzyme (as discussed above); so, improved NO_3_^-^ content improves NR activity.

The NH_4_^+^ formed by sequential reduction of NO_3_^-^ is rapidly assimilated into organic compounds (amino acids) by ammonia assimilating enzymes, i.e., GS and GOGAT. The substantial decrease in GS and GOGAT activity caused by As might disturbed the C/N equilibrium, which negatively regulated the growth of test plant (**Figures 1A,B**). This decrease in GS activity could be due to the downregulation of aminotrasferases enzymes (involved in glycolysis, amino acid metabolism, photorespiration, and N used efficiency; Good et al., 2007), which would have resulted into lesser N content in *Brassica* seedlings as reported by Dixit et al. (2015). The decrease in GOGAT activity might be due to oxidative modifications credited by excessive ROS generation, under As treatment (**Figure 6**). Hence, as an outcome of decreased GS and GOGAT activities, a sharp increase in NH_4_^+^ content in root and leaf tissues of test seedlings under As stress was observed, which suggests impairment in NH_4_^+^ assimilation process. The decreased NO_3_^-^ and increased NH_4_^+^ content justifies the reason for increased NH_4_^+^/NO_3_^-^ ratio (**Figure 4**). The higher ratio of NH_4_^+^ to NO_3_^-^ in *B. juncea* was caused mainly by reduction in NO_3_^-^ uptake and NH_4_^+^ assimilation rate rather than NH_4_^+^ uptake, which could be justified by higher NH_4_^+^ accumulation and decline in GS activity. However, S and Ca application either alone or in combination to As stressed *Brassica* seedlings caused less inhibitory effect on GS and GOGAT activities (**Figure 6**), which could be explained as: (i) proper incorporation of NH_4_^+^ into glutamate as less NH_4_^+^ accumulation was found in root and leaves in comparison to As treatment alone (**Figure 4**) and (ii) less ROS accumulation that thereby causes minor alteration in active sites of these enzymes.

Moreover, when NH_4_^+^ gets hyper accumulated in plants, it directly incorporated into glutamate by triggering the GDH (aminating) enzyme and its activity was sharply increased at tested doses of As (**Figures 6E,F**). This increase in GDH activity (with high Km) might be the plants’ compensation for the inhibited GS and GOGAT activities. Thus, increased GDH activity might have helped in relieving the pressure caused by accumulation of NH_4_^+^ (Skopelitis et al., 2006); so, increased GDH activity could be a selective advantage to carry on NH_4_^+^ assimilation under As stress in test seedlings.

In the present study, the decrease in protein content subjected to As stress (**Figure 7**) might result from: (i) enhanced protein degradation rate as a result of increased protease activity (Stoeva and Bineva, 2003), (ii) As may reacts with –SH group of enzymes and proteins (Ullrich-Eberius et al., 1989) and (iii) decline in amino acid content as an outcome of considerable inhibition in GS and GOGAT activities in *Brassica* seedlings (**Figure 6**). Carbohydrate content might be decreased as: (i) Chl *a* contents are directly associated with carbohydrate production, so decreased Chl *a* contents might have decreased the carbohydrate content (**Figure 7**; Choudhury et al., 2011) and (ii) decrease in photosynthetic rate (**Figure 1**). Contrary to this, S and Ca improved the protein and carbohydrate contents in As stressed plant. The role of S and Ca in enhancing protein content may be attributed to: (i) enhanced protein synthesis as S is involved in di-sulfide (S–S) linkage of protein, (ii) decrease in proteolysis, (iii) lowering of enzyme denaturation processes, that occurs during abiotic stress (Levitt, 1980), and (iv) improvement in amino acid influx due to improvement in GS-GOGAT pathway (**Figure 6**). On the other hand, the increase in carbohydrate content induced by S and Ca have also been reported in chickpea plant grown under Cd stress (Ahmad et al., 2016) and flax plant grown under newly reclaimed sandy soils (Bakry et al., 2015). The explanation for this increment in carbohydrate content could be the improved photosynthetic efficiency (**Figure 1**) that might enhance the biosynthesis of carbohydrates.

## Conclusion

From the above study, it is concluded that *B. juncea* L. seedlings, the third highest oil seed crop of the world, is suffering from As toxicity especially in South-East Asia, where As contamination is at alarming stage. Results clearly revealed that As negatively regulates the growth by interfering with the two key metabolic processes, i.e., photosynthesis and nitrogen metabolism (by inhibiting enzyme activities) as evident from higher As/S and As/Ca ratio in root followed by shoots/leaves. Contrastingly, S and/or Ca application following As treatment provides significant protection to *Brassica* seedlings by improving the photosynthetic performance and activities of enzymes of nitrogen metabolism. Furthermore, in the present study, the more pronounced role of S+Ca was observed in As-challenged *Brassica* seedlings.

## Author Contributions

SP designed the experiment. RS and PP performed the experiment. SP, RS, and PP wrote and finalized the manuscript.

## Conflict of Interest Statement

The authors declare that the research was conducted in the absence of any commercial or financial relationships that could be construed as a potential conflict of interest.
